# Poly[tetra-μ_1,1_-azido-bis­(μ_2_-pyrimidine-2-carboxyl­ato)tricopper(II)]

**DOI:** 10.1107/S1600536810027030

**Published:** 2010-07-21

**Authors:** Jiong-Peng Zhao, Fu-Chen Liu

**Affiliations:** a School of Chemistry and Chemical Engineering, Tianjin University of Technology, Tianjin 300191, People’s Republic of China

## Abstract

In the title compound, [Cu_3_(C_5_H_3_N_2_O_2_)_2_(N_3_)_4_]_*n*_, one of the Cu^II^ atoms lies on an inversion centre and is octa­hedrally coordinated by two bidentate chelating pyrimidine-2-carboxyl­ate ligands and two azide anions, each of which gives an *N*:*N*-bridge to the second inversion-related Cu^II^ centre in the formula unit. The second Cu^II^ atom is five-coordinated with a distorted square-pyramidal coordination sphere comprising a single bidentate chelating pyrimidine-2-carboxyl­ate anion and three azide N anions, two of which doubly bridge centrosymmetric Cu^II^ centres, giving a two-dimensional network structure extending parallel to (010).

## Related literature

Copper azide complexes have attracted much attention in recent years because the azide anions can mediate magnetic inter­actions effectively between the copper ions, see: Zhao *et al.* (2009 ▶). The structures of the complexes are dependant on the co-ligand and conditions employed in the synthesis, see: Zeng *et al.* (2009 ▶). For azide complexes with 2,2′-bipyrimidine or oxalate as co-ligands, see: Cortes *et al.* (1996 ▶); Escuer *et al.* (1994 ▶) and for an azide complex with a pyrimidine-2-carboxyl­ate ligand, see: Suarez-Varela *et al.* (2008 ▶).
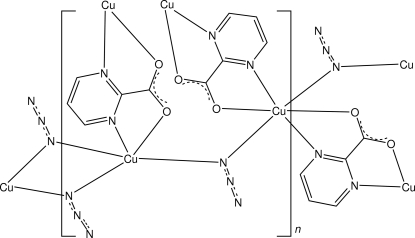


## Experimental

### 

#### Crystal data

[Cu_3_(C_5_H_3_N_2_O_2_)_2_(N_3_)_4_]
*M* *_r_* = 604.96
Monoclinic, 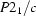

*a* = 7.4743 (15) Å
*b* = 14.997 (3) Å
*c* = 9.479 (4) Å
β = 122.31 (2)°
*V* = 898.0 (5) Å^3^
*Z* = 2
Mo *K*α radiation
μ = 3.59 mm^−1^
*T* = 293 K
0.20 × 0.18 × 0.18 mm

#### Data collection

Rigaku SCXmini CCD diffractometer
Absorption correction: multi-scan (*ABSCOR*; Higashi, 1995 ▶) *T*_min_ = 0.625, *T*_max_ = 1.000
7028 measured reflections
1572 independent reflections
1305 reflections with *I* > 2σ(*I*)
*R*_int_ = 0.061

#### Refinement

*R*[*F* ^2^ > 2σ(*F* ^2^)] = 0.052
*wR*(*F* ^2^) = 0.134
*S* = 1.24
1572 reflections
151 parameters
H-atom parameters constrained
Δρ_max_ = 0.71 e Å^−3^
Δρ_min_ = −0.46 e Å^−3^

### 

Data collection: *SCXmini Benchtop Crystallography System Software* (Rigaku, 2006 ▶); cell refinement: *PROCESS-AUTO* (Rigaku, 1998 ▶); data reduction: *PROCESS-AUTO*; program(s) used to solve structure: *SHELXS97* (Sheldrick, 2008 ▶); program(s) used to refine structure: *SHELXL97* (Sheldrick, 2008 ▶); molecular graphics: *ORTEPIII* (Burnett & Johnson, 1996 ▶) and *PLATON* (Spek, 2009 ▶); software used to prepare material for publication: *SHELXTL* (Sheldrick, 2008 ▶).

## Supplementary Material

Crystal structure: contains datablocks global, I. DOI: 10.1107/S1600536810027030/zs2045sup1.cif

Structure factors: contains datablocks I. DOI: 10.1107/S1600536810027030/zs2045Isup2.hkl

Additional supplementary materials:  crystallographic information; 3D view; checkCIF report

## Figures and Tables

**Fig. 1.**
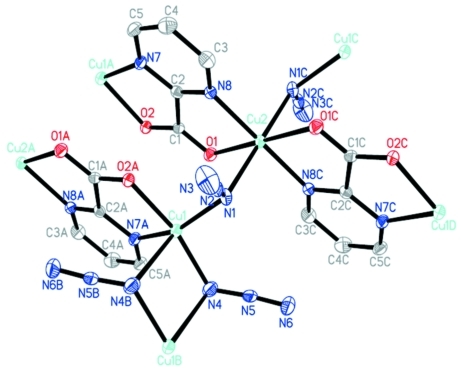
The coordination mode and linkage of the metal ions and ligands in (I). Ellipsoids are drawn at the 30% probability level. H atoms have been omitted for clarity. [Symmetry codes: (A) -x + 2, -y, -z; (B) -x + 1, -y, -z - 1; (C) -x + 1, -y, -z; (D) x - 1, y, z].

**Fig. 2.**
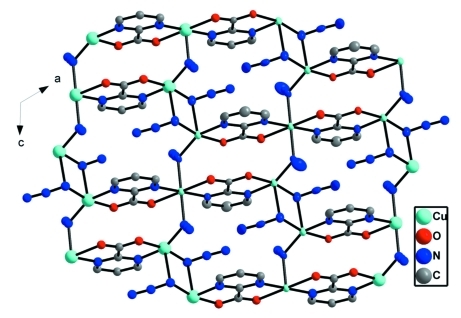
The two-dimensional network structure of (I).

**Fig. 3.**
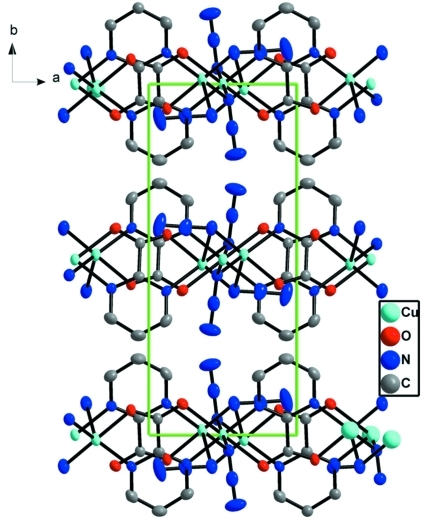
The packing mode of the complex in the unit cell.

## supplementary crystallographic information

### Comment

Copper azide complexes have attracted much attention in recent years because the azide anions can mediate magnetic interactions effectively between the copper ions (Zhao *et al.*, 2009). The structures of those complexes are dependant on the co-ligand and conditions employed in the synthesis (Zeng *et al.*, 2009). Some azide complexes with 2,2'-bipyrimidine or oxalate as co-ligands have been reported (Cortes *et al.*, 1996); Escuer *et al.*, 1994). The pyrimidine-2-carboxylate ligand can be considered as the combination of 2,2'-bipyrimidine and oxalate, and a new metal azide complex with it as ligand has been reported (Suarez-Varela *et al.*, 2008). In this work we report a new copper(II) azide complex with pyrimidine-2-carboxylate as co-ligand, [Cu_3_(C_5_H_3_N_2_O_2_)_2_(N_3_)_4_]*_n_* (I), prepared under hydrothermal conditions and its structure is reported here.

In the asymmetric units of the title compound, there are one and a half copper(II) cations, two azido anions and two pyrimidine-2-carboxylate ligands (Fig. 1). One of the cations (Cu2) lies on an inversion centre and is octahedrally coordinated by two bidentate chelate pyrimidine-2-carboxylato-N,O ligands and two azido anions, each giving an N bridge to the inversion-related Cu1 centres in the formula unit [Cu2—Cu1, 3.4652 (14) Å]. A second weak contact between a carboxyl O (O1) to Cu1 is also present [Cu1···O1, 2.950 (4) Å] but is too long to be considered a bridging (Cu–O) bond. The coordination sphere about Cu1 is five-coordinate with a distorted square pyramidal coordination sphere comprising a single bidentate chelate pyrimidine-2-carboxylate anion and three azido N anions, one bridging to Cu2, the other two giving double N bridges to centrosymmetrically related Cu centres [Cu1—Cu1^iii^, 3.329 (2) Å] [symmetry code: (iii) *x* -1, *y*, *z*]. Structure extension results in a two-dimensional network (Figs. 2, 3).

### Experimental

A mixture of copper(II) nitrate (1.5mmol) and sodium azide (2 mmol), and pyrimidine-2-carboxylic acid (0.5 mmol) in 10 ml of water was sealed in a Teflon-lined stainless-steel Parr bomb that was heated at 413 K for 48 h. Black crystals of the title complex were collected after the bomb was allowed to cool to room temperature (yield 20% based on Cu). Caution: azides may be explosive: although we have had no problems in this work, only small quantities should be prepared and should be handled with great caution.

### Refinement

Hydrogen atoms were included in calculated positions and treated as riding on their parent C atoms with C—H = 0.93Å and *U*_iso_(H) = 1.2*U*_eq_(C).

### Crystal data

**Table:**

[Cu_3_(C_5_H_3_N_2_O_2_)_2_(N_3_)_4_]	*F*(000) = 594
*M**_r_* = 604.96	*D*_x_ = 2.237 Mg m^−^^3^
Monoclinic, *P*2_1_/*c*	Mo *K*α radiation, λ = 0.71073 Å
Hall symbol: -P 2ybc	Cell parameters from 7433 reflections
*a* = 7.4743 (15) Å	θ = 3.2–27.5°
*b* = 14.997 (3) Å	µ = 3.59 mm^−^^1^
*c* = 9.479 (4) Å	*T* = 293 K
β = 122.31 (2)°	Block, black
*V* = 898.0 (5) Å^3^	0.20 × 0.18 × 0.18 mm
*Z* = 2	

### Data collection

**Table:**

Rigaku SCXmini CCD diffractometer	1572 independent reflections
Radiation source: fine-focus sealed tube	1305 reflections with *I* > 2σ(*I*)
graphite	*R*_int_ = 0.061
ω scans	θ_max_ = 25.0°, θ_min_ = 3.2°
Absorption correction: multi-scan (*ABSCOR*; Higashi, 1995)	*h* = −8→8
*T*_min_ = 0.625, *T*_max_ = 1.000	*k* = −17→17
7028 measured reflections	*l* = −11→11

### Refinement

**Table:**

Refinement on *F*^2^	Primary atom site location: structure-invariant direct methods
Least-squares matrix: full	Secondary atom site location: difference Fourier map
*R*[*F*^2^ > 2σ(*F*^2^)] = 0.052	Hydrogen site location: inferred from neighbouring sites
*wR*(*F*^2^) = 0.134	H-atom parameters constrained
*S* = 1.24	*w* = 1/[σ^2^(*F*_o_^2^) + (0.0543*P*)^2^ + 2.0516*P*] where *P* = (*F*_o_^2^ + 2*F*_c_^2^)/3
1572 reflections	(Δ/σ)_max_ < 0.001
151 parameters	Δρ_max_ = 0.71 e Å^−^^3^
0 restraints	Δρ_min_ = −0.46 e Å^−^^3^

### Special details

**Table:**

Geometry. Bond distances, angles etc. have been calculated using the rounded fractional coordinates. All su's are estimated from the variances of the (full) variance-covariance matrix. The cell esds are taken into account in the estimation of distances, angles and torsion angles
Refinement. Refinement of *F*^2^ against ALL reflections. The weighted *R*-factor *wR* and goodness of fit *S* are based on *F*^2^, conventional *R*-factors *R* are based on *F*, with *F* set to zero for negative *F*^2^. The threshold expression of *F*^2^ > σ(*F*^2^) is used only for calculating *R*-factors(gt) *etc*. and is not relevant to the choice of reflections for refinement. *R*-factors based on *F*^2^ are statistically about twice as large as those based on *F*, and *R*- factors based on ALL data will be even larger.

### Fractional atomic coordinates and isotropic or equivalent isotropic displacement parameters (Å^2^)

**Table:**

	*x*	*y*	*z*	*U*_iso_*/*U*_eq_	
Cu1	0.64025 (11)	−0.01316 (5)	−0.29370 (10)	0.0259 (3)	
Cu2	0.50000	0.00000	0.00000	0.0221 (3)	
O1	0.7728 (6)	−0.0882 (3)	0.0359 (5)	0.0278 (12)	
O2	1.1215 (6)	−0.0703 (3)	0.1528 (5)	0.0234 (12)	
N1	0.4612 (8)	0.0444 (4)	−0.2223 (7)	0.0279 (17)	
N2	0.4377 (9)	0.1253 (4)	−0.2455 (7)	0.0344 (19)	
N3	0.4134 (13)	0.2001 (5)	−0.2695 (10)	0.063 (3)	
N4	0.4241 (9)	−0.0895 (4)	−0.4673 (7)	0.0365 (19)	
N5	0.2454 (9)	−0.0933 (4)	−0.4977 (6)	0.035 (2)	
N6	0.0719 (11)	−0.0987 (6)	−0.5367 (8)	0.064 (3)	
N7	1.1181 (8)	0.0896 (3)	0.2648 (6)	0.0245 (17)	
N8	0.7462 (8)	0.0827 (3)	0.1309 (6)	0.0224 (17)	
C1	0.9407 (9)	−0.0464 (4)	0.1173 (7)	0.0214 (17)	
C2	0.9320 (9)	0.0481 (4)	0.1760 (7)	0.0221 (17)	
C3	0.7414 (11)	0.1682 (4)	0.1722 (9)	0.033 (2)	
C4	0.9286 (12)	0.2167 (5)	0.2647 (9)	0.038 (3)	
C5	1.1161 (11)	0.1746 (4)	0.3094 (8)	0.032 (2)	
H3A	0.61250	0.19510	0.13880	0.0390*	
H4A	0.92720	0.27550	0.29530	0.0460*	
H5A	1.24310	0.20550	0.37130	0.0390*	

### Atomic displacement parameters (Å^2^)

**Table:**

	*U*^11^	*U*^22^	*U*^33^	*U*^12^	*U*^13^	*U*^23^
Cu1	0.0184 (4)	0.0326 (5)	0.0258 (5)	−0.0030 (3)	0.0112 (4)	−0.0039 (3)
Cu2	0.0168 (6)	0.0274 (6)	0.0215 (6)	−0.0014 (4)	0.0099 (5)	−0.0009 (4)
O1	0.026 (2)	0.026 (2)	0.030 (2)	−0.0038 (19)	0.014 (2)	−0.0047 (19)
O2	0.018 (2)	0.028 (2)	0.023 (2)	−0.0004 (17)	0.0102 (18)	−0.0010 (18)
N1	0.025 (3)	0.031 (3)	0.034 (3)	−0.001 (2)	0.020 (3)	0.001 (3)
N2	0.029 (3)	0.048 (4)	0.035 (3)	−0.001 (3)	0.023 (3)	−0.001 (3)
N3	0.091 (6)	0.032 (4)	0.092 (6)	0.014 (4)	0.066 (5)	0.011 (4)
N4	0.026 (3)	0.045 (4)	0.035 (3)	−0.003 (3)	0.014 (3)	−0.010 (3)
N5	0.035 (4)	0.050 (4)	0.018 (3)	−0.012 (3)	0.012 (3)	−0.002 (3)
N6	0.034 (4)	0.114 (7)	0.040 (4)	−0.025 (4)	0.018 (3)	−0.001 (4)
N7	0.021 (3)	0.024 (3)	0.026 (3)	−0.005 (2)	0.011 (2)	−0.003 (2)
N8	0.021 (3)	0.025 (3)	0.022 (3)	−0.001 (2)	0.012 (2)	−0.002 (2)
C1	0.021 (3)	0.026 (3)	0.016 (3)	−0.001 (3)	0.009 (3)	0.000 (3)
C2	0.027 (3)	0.020 (3)	0.022 (3)	0.000 (3)	0.015 (3)	0.002 (2)
C3	0.035 (4)	0.029 (4)	0.037 (4)	0.007 (3)	0.021 (3)	0.002 (3)
C4	0.047 (5)	0.025 (4)	0.041 (4)	0.000 (3)	0.022 (4)	−0.004 (3)
C5	0.030 (4)	0.035 (4)	0.026 (4)	−0.011 (3)	0.012 (3)	−0.004 (3)

### Geometric parameters (Å, °)

**Table:**

Cu1—N1	1.991 (7)	N2—N3	1.140 (10)
Cu1—N4	1.946 (6)	N4—N5	1.208 (10)
Cu1—N4^i^	2.563 (6)	N5—N6	1.146 (12)
Cu1—O2^ii^	1.995 (5)	N7—C2	1.334 (9)
Cu1—N7^ii^	2.030 (6)	N7—C5	1.346 (8)
Cu2—O1	2.298 (5)	N8—C2	1.321 (10)
Cu2—N1	2.077 (6)	N8—C3	1.347 (8)
Cu2—N8	2.006 (6)	C1—C2	1.537 (9)
Cu2—O1^iii^	2.298 (5)	C3—C4	1.394 (12)
Cu2—N1^iii^	2.077 (6)	C4—C5	1.381 (13)
Cu2—N8^iii^	2.006 (5)	C3—H3A	0.9300
O1—C1	1.236 (8)	C4—H4A	0.9300
O2—C1	1.258 (9)	C5—H5A	0.9300
N1—N2	1.229 (8)		
			
N1—Cu1—N4	97.9 (3)	Cu1—N1—N2	115.2 (5)
N1—Cu1—N4^i^	101.4 (2)	Cu2—N1—N2	115.3 (5)
O2^ii^—Cu1—N1	91.3 (2)	N1—N2—N3	178.9 (8)
N1—Cu1—N7^ii^	155.5 (2)	Cu1—N4—N5	122.8 (5)
N4—Cu1—N4^i^	85.8 (2)	Cu1—N4—Cu1^i^	94.2 (2)
O2^ii^—Cu1—N4	167.4 (2)	Cu1^i^—N4—N5	99.0 (4)
N4—Cu1—N7^ii^	93.4 (3)	N4—N5—N6	175.6 (7)
O2^ii^—Cu1—N4^i^	83.89 (19)	C2—N7—C5	117.2 (6)
N4^i^—Cu1—N7^ii^	101.0 (2)	Cu1^ii^—N7—C2	112.0 (4)
O2^ii^—Cu1—N7^ii^	81.5 (2)	Cu1^ii^—N7—C5	130.6 (5)
O1—Cu2—N1	88.1 (2)	Cu2—N8—C2	114.5 (4)
O1—Cu2—N8	79.4 (2)	Cu2—N8—C3	127.6 (6)
O1—Cu2—O1^iii^	180.00	C2—N8—C3	117.8 (6)
O1—Cu2—N1^iii^	91.9 (2)	O1—C1—O2	127.6 (6)
O1—Cu2—N8^iii^	100.6 (2)	O1—C1—C2	117.9 (6)
N1—Cu2—N8	90.8 (2)	O2—C1—C2	114.4 (6)
O1^iii^—Cu2—N1	91.9 (2)	N7—C2—N8	125.6 (6)
N1—Cu2—N1^iii^	180.00	N7—C2—C1	115.3 (6)
N1—Cu2—N8^iii^	89.2 (2)	N8—C2—C1	119.1 (6)
O1^iii^—Cu2—N8	100.6 (2)	N8—C3—C4	120.4 (8)
N1^iii^—Cu2—N8	89.2 (2)	C3—C4—C5	117.9 (7)
N8—Cu2—N8^iii^	180.00	N7—C5—C4	121.1 (7)
O1^iii^—Cu2—N1^iii^	88.1 (2)	N8—C3—H3A	120.00
O1^iii^—Cu2—N8^iii^	79.4 (2)	C4—C3—H3A	120.00
N1^iii^—Cu2—N8^iii^	90.8 (2)	C3—C4—H4A	121.00
Cu2—O1—C1	109.0 (4)	C5—C4—H4A	121.00
Cu1^ii^—O2—C1	116.6 (4)	N7—C5—H5A	119.00
Cu1—N1—Cu2	116.8 (3)	C4—C5—H5A	119.00
			
O1—Cu1—N1—N2	136.0 (5)	N8—Cu2—N1—Cu1	84.3 (3)
N4—Cu1—N1—Cu2	103.7 (3)	N8—Cu2—N1—N2	−55.7 (6)
N4—Cu1—N1—N2	−116.3 (5)	O1^iii^—Cu2—N1—Cu1	−175.1 (3)
N4^i^—Cu1—N1—Cu2	−169.0 (3)	O1^iii^—Cu2—N1—N2	44.9 (6)
N4^i^—Cu1—N1—N2	−28.9 (5)	N8^iii^—Cu2—N1—Cu1	−95.7 (3)
O2^ii^—Cu1—N1—Cu2	−84.9 (3)	N8^iii^—Cu2—N1—N2	124.3 (6)
O2^ii^—Cu1—N1—N2	55.1 (5)	O1—Cu2—N8—C2	−2.2 (4)
N7^ii^—Cu1—N1—Cu2	−12.9 (8)	O1—Cu2—N8—C3	175.8 (6)
N7^ii^—Cu1—N1—N2	127.2 (6)	N1—Cu2—N8—C2	−90.1 (5)
O1—Cu1—N4—N5	72.4 (6)	N1—Cu2—N8—C3	87.9 (6)
O1—Cu1—N4—Cu1^i^	175.86 (15)	O1^iii^—Cu2—N8—C2	177.8 (4)
N1—Cu1—N4—N5	−2.6 (6)	O1^iii^—Cu2—N8—C3	−4.3 (6)
N1—Cu1—N4—Cu1^i^	100.9 (2)	N1^iii^—Cu2—N8—C2	89.9 (5)
N4^i^—Cu1—N4—N5	−103.5 (6)	N1^iii^—Cu2—N8—C3	−92.2 (6)
N4^i^—Cu1—N4—Cu1^i^	0.0 (2)	Cu1—O1—C1—O2	−91.6 (6)
N7^ii^—Cu1—N4—N5	155.7 (6)	Cu1—O1—C1—C2	84.7 (5)
N7^ii^—Cu1—N4—Cu1^i^	−100.8 (2)	Cu2—O1—C1—O2	−175.2 (5)
N1—Cu1—N4^i^—Cu1^i^	−97.2 (3)	Cu2—O1—C1—C2	1.2 (6)
N1—Cu1—N4^i^—N5^i^	138.6 (4)	Cu1^ii^—O2—C1—O1	−178.1 (5)
N4—Cu1—N4^i^—Cu1^i^	0.0 (3)	Cu1^ii^—O2—C1—C2	5.5 (6)
N4—Cu1—N4^i^—N5^i^	−124.2 (5)	C5—N7—C2—N8	−1.4 (9)
O1—Cu1—O2^ii^—C1^ii^	87.9 (4)	C5—N7—C2—C1	175.8 (5)
N1—Cu1—O2^ii^—C1^ii^	160.8 (4)	Cu1^ii^—N7—C2—N8	−176.8 (5)
O1—Cu1—N7^ii^—C2^ii^	−84.5 (4)	Cu1^ii^—N7—C2—C1	0.4 (6)
O1—Cu1—N7^ii^—C5^ii^	90.1 (6)	C2—N7—C5—C4	0.0 (10)
N1—Cu1—N7^ii^—C2^ii^	−75.9 (7)	Cu1^ii^—N7—C5—C4	174.4 (5)
N1—Cu1—N7^ii^—C5^ii^	98.7 (7)	Cu2—N8—C2—N7	−179.3 (5)
N4—Cu1—N7^ii^—C2^ii^	166.6 (4)	Cu2—N8—C2—C1	3.6 (7)
N4—Cu1—N7^ii^—C5^ii^	−18.8 (6)	C3—N8—C2—N7	2.6 (9)
N1—Cu2—O1—Cu1	−2.98 (18)	C3—N8—C2—C1	−174.6 (6)
N1—Cu2—O1—C1	91.6 (4)	Cu2—N8—C3—C4	179.9 (5)
N8—Cu2—O1—Cu1	−94.14 (17)	C2—N8—C3—C4	−2.2 (10)
N8—Cu2—O1—C1	0.4 (4)	O1—C1—C2—N7	179.3 (5)
N1^iii^—Cu2—O1—Cu1	177.02 (18)	O1—C1—C2—N8	−3.3 (8)
N1^iii^—Cu2—O1—C1	−88.4 (4)	O2—C1—C2—N7	−3.9 (8)
N8^iii^—Cu2—O1—Cu1	85.86 (17)	O2—C1—C2—N8	173.5 (5)
N8^iii^—Cu2—O1—C1	−179.6 (4)	N8—C3—C4—C5	1.0 (11)
O1—Cu2—N1—Cu1	4.9 (3)	C3—C4—C5—N7	0.2 (11)
O1—Cu2—N1—N2	−135.1 (6)		

Symmetry codes: (i) −*x*+1, −*y*, −*z*−1; (ii) −*x*+2, −*y*, −*z*; (iii) −*x*+1, −*y*, −*z*.
